# Myosteatosis Significantly Predicts Persistent Dyspnea and Mobility Problems in COVID-19 Survivors

**DOI:** 10.3389/fnut.2022.846901

**Published:** 2022-04-08

**Authors:** Rebecca De Lorenzo, Anna Palmisano, Antonio Esposito, Chiara Gnasso, Valeria Nicoletti, Riccardo Leone, Davide Vignale, Elisabetta Falbo, Marica Ferrante, Marta Cilla, Cristiano Magnaghi, Sabina Martinenghi, Giordano Vitali, Alessio Molfino, Patrizia Rovere-Querini, Maurizio Muscaritoli, Caterina Conte

**Affiliations:** ^1^Division of Immunology, Transplantation and Infectious Diseases, IRCCS San Raffaele Scientific Institute, Milan, Italy; ^2^School of Medicine, Vita-Salute San Raffaele University, Milan, Italy; ^3^Clinical and Experimental Radiology Unit, Experimental Imaging Center, IRCCS San Raffaele Scientific Institute, Milan, Italy; ^4^Department of Translational and Precision Medicine, Sapienza University of Rome, Rome, Italy; ^5^Department of Human Sciences and Promotion of the Quality of Life, San Raffaele Roma Open University, Rome, Italy; ^6^Department of Endocrinology, Nutrition and Metabolic Diseases, IRCCS MultiMedica, Milan, Italy

**Keywords:** myosteatosis, SARS-CoV-2, sarcopenia, long-COVID, obesity, skeletal muscle, dyspnea

## Abstract

**Background:**

Persistent symptoms including dyspnea and functional impairment are common in COVID-19 survivors. Poor muscle quality (myosteatosis) associates with poor short-term outcomes in COVID-19 patients. The aim of this observational study was to assess the relationship between myosteatosis diagnosed during acute COVID-19 and patient-reported outcomes at 6 months after discharge.

**Methods:**

Myosteatosis was diagnosed based on CT-derived skeletal muscle radiation attenuation (SM-RA) measured during hospitalization in 97 COVID-19 survivors who had available anthropometric and clinical data upon admission and at the 6-month follow-up after discharge. Dyspnea in daily activities was assessed using the modified Medical Research Council (mMRC) scale for dyspnea. Health-related quality of life was measured using the European quality of life questionnaire three-level version (EQ-5D-3L).

**Results:**

Characteristics of patients with (lowest sex- and age-specific tertile of SM-RA) or without myosteatosis during acute COVID-19 were similar. At 6 months, patients with myosteatosis had greater rates of obesity (48.4 vs. 27.7%, *p* = 0.046), abdominal obesity (80.0 vs. 47.6%, *p* = 0.003), dyspnea (32.3 vs. 12.5%, *p* = 0.021) and mobility problems (32.3 vs. 12.5%, *p* = 0.004). Myosteatosis diagnosed during acute COVID-19 was the only significant predictor of persistent dyspnea (OR 3.19 [95% C.I. 1.04; 9.87], *p* = 0.043) and mobility problems (OR 3.70 [95% C.I. 1.25; 10.95], *p* = 0.018) at 6 months at logistic regression adjusted for sex, age, and BMI.

**Conclusion:**

Myosteatosis diagnosed during acute COVID-19 significantly predicts persistent dyspnea and mobility problems at 6 months after hospital discharge independent of age, sex, and body mass.

**Clinical Trial Registration:**

[www.ClinicalTrials.gov], identifier [NCT04318366].

## Introduction

Coronavirus disease 2019 (COVID-19) poses a severe burden on survivors, with clinically relevant nutritional and functional impairments. At hospital discharge, more than 70% of patients who survive critical COVID-19 report problems in mobility and in conducting their usual activities (1). At 6 months after hospital discharge, a significant proportion of COVID-19 survivors have impaired functional status (2) and persistent symptoms including dyspnea, fatigue, and muscle weakness (3). Patients with more severe illness during the acute phase are those with more troublesome sequelae (4). However, even patients with mild disease experience persisting symptoms after SARS-CoV-2 infection (5, 6). Few studies have investigated factors associated with symptom persistence in the long-term. Frailty, multiple symptoms at disease onset, female sex, endothelial dysfunction, overweight/obesity, and pre-existing comorbidities have been suggested as potential predictors (4, 7–9). Reduced muscle mass and quality might also contribute to persistent functional impairment. In non-COVID-19 critically ill patients, pre-existing low muscle mass independently predicts poor functional status at 12 months after intra−abdominal sepsis (10). Muscle quality is even more related to muscle function or strength as compared with muscle mass (11). The term *muscle quality* generally refers to the ability of skeletal muscle to effectively perform its functions, the most prominent being force production. Muscle quality depends on several determinants, including fiber type and distribution, muscle architecture, neuromuscular activation, the amount of fibrous tissue and the extent of fat infiltration, i.e., myosteatosis (12). The latter has emerged as an important biomarker and a priority research focus, being an independent risk factor for metabolic dysfunction, hip fractures, disability, hospitalization, mortality, and poor outcomes in severe illness and surgery (13, 14). Computed tomography (CT)-derived skeletal muscle radiodensity, also known as skeletal muscle radiation attenuation (SM-RA), is a reliable marker of myosteatosis (15). Low CT-derived SM-RA, which indicates myosteatosis, is independently associated with higher 6-month mortality in critically ill patients (16). In surgical patients, low SM-RA predicts postoperative complications or mortality (17–21), being a more reliable predictor than muscle size (20, 21). Furthermore, low trunk SM-RA is strongly associated with reduced functional capacity in both inpatients and outpatients (22–24).

Recent evidence indicates that myosteatosis was diagnosed by opportunistic CT-derived SM-RA associates with poor short-term outcomes in COVID-19 patients (25–28). It is not known whether poor muscle quality, as reflected by the presence of myosteatosis, influences clinical outcomes of COVID-19 survivors in the medium term. Therefore, the aim of this study was to assess the relationship between myosteatosis detected during acute COVID-19 and patient reported outcomes at 6 months after discharge.

## Materials and Methods

### Study Design

This analysis is part of the COVID-BioB study, a large observational investigation performed at San Raffaele University Hospital in Milan, Italy. The study protocol was approved by the IRCCS San Raffaele Hospital Ethics Committee (protocol no. 34/int/2020) and was registered on ClinicalTrials.gov (NCT04318366). The study was conducted in accordance with the World Medical Association’s Declaration of Helsinki. All patients provided a signed informed consent. Full description of patient management and clinical protocols were previously published (29, 30). The reporting of this study conforms to the Strengthening the Reporting of Observational Studies in Epidemiology (STROBE) Statement for cohort studies (31).

### Participants

All patients aged ≥ 18 years admitted to the Emergency Department (ED) at San Raffaele University Hospital with confirmed SARS-CoV-2 infection were consecutively enrolled in the COVID-BioB study. Confirmed infection was defined as positive real-time reverse-transcriptase polymerase chain reaction (RT-PCR) from a nasal and/or throat swab together with signs, symptoms, and radiological findings suggestive of COVID-19 pneumonia. Only COVID-19 survivors hospitalized during the first pandemic wave who underwent a CT scan and had available anthropometric data upon admission and at the 6-month follow-up were included in the present analysis. Patients admitted for other reasons and subsequently diagnosed with superimposed SARS-CoV-2 infection were excluded.

### Assessments

Data were collected from medical chart review or directly by patient interview and entered in a dedicated electronic case record form (eCRF) specifically developed for the COVID-BioB study. Prior to the analysis, data were cross-checked with medical charts and verified by data managers and clinicians for accuracy. The following variables were collected for all patients: age, sex, body mass index [BMI, calculated as the ratio of weight in kilograms (kg) divided by height in squared meters], laboratory parameters on hospital admission, comorbidities (including history of hypertension, diabetes mellitus, ischaemic heart disease, and active malignancy), length of stay (LoS) and treatment intensity [admission to the intensive care unit (ICU)]. The 6-month follow-up visit included a complete internal medicine assessment (collection of medical history, physical examination), and measurement of anthropometrics (body weight measured to the nearest 0.1 kg using a balance beam scale, height measured to the nearest 0.1 cm using a wall-mounted stadiometer, waist circumference measurements taken around the abdomen at the level of the umbilicus). Abdominal obesity was defined as a waist circumference ≥ 88 cm in women and ≥ 102 cm in men (32).

Dyspnea in daily activities was assessed using the modified Medical Research Council (mMRC) scale for dyspnea. Patients with a mMRC score ≥ 2 were classified as having dyspnea (33, 34).

Health-related quality of life was measured using the European quality of life questionnaire three-level version (EQ-5D-3L) (35). The EQ-5D-3L comprises five dimensions (mobility, self-care, usual activities, pain/discomfort, and anxiety/depression) and a visual analog scale (EQ VAS). Each dimension has 3 levels, labeled 1–3: no problems, some problems, and extreme problems. For the present analysis, the EQ-5D levels were dichotomized into “no problems” (level 1) and “any problems” (levels 2 and 3). The EQ VAS records the patient’s self-rated health on a vertical visual analog scale where the endpoints are labeled “best imaginable health state” and “worst imaginable health state.”

### CT Scan Protocol and Image Analysis

Non-contrast chest CT scans were performed in a dedicated suite easily accessible *via* assigned elevators and paths, on a 64-slice scanner (LightSpeed V CT, GE Healthcare) in supine position, during inspiratory breath hold. CT scan parameters were as follows: 120 kV tube voltage, automatic tube current modulation 150–550 mA, 0⋅4 s rotation time, pitch 1⋅375 mm/rot, 64 × 0⋅625 mm detector collimation. Images were reconstructed at 1⋅25 and 3 mm slice thickness with sharp and medium-soft kernel, respectively, for lung and mediastinum evaluation. The lung and mediastinal window width and level were set as 1,500/–700 Hounsfield Units (HU) and 350/40 HU, respectively. Mean dose length product was 438 ± 153 mGy⋅cm.

SM-RA was measured directly analyzing CT images on the picture archiving and communication system (PACS) on axial images with mediastinal filter reconstruction. Cross-sectional areas (CSA) of the paravertebral skeletal muscle mass on both sides of the spine including the *erector spinae*, *longissimus thoracis*, *spinalis thoracis*, and *iliocostalis lumborum* muscles were manually drawn at T11-T12 level. To estimate muscle mass, the paravertebral skeletal muscle index (pSMI) was calculated as the bilateral cross-sectional area of paravertebral skeletal muscles normalized by the height squared (cm^2^/m^2^) (36, 37). The SM-RA values relative to each ROI were averaged to obtain a single value for each patient. The average SM-RA values reflect the composition of muscle, with lower mean values due to the intrinsic difference in HU values between muscle (-29 to + 150 HU) (38) and fat (−30 to −190 HU) (39) indicating higher fat content. Therefore, the lower the SM-RA, the higher the degree of myosteatosis (14). As univocal cut-off values based on paraspinal SM-RA for diagnosing thoracic myosteatosis are not available (13), we identified our own cut-off values based on tertiles stratified by age (≥ or < 60 years) and sex. A similar approach has been adopted by other authors for samples of similar size (28, 40). Cutoff values for defining myosteatosis were set at the lowest tertiles of SM-RA.

### Statistical Analysis

Descriptive statistics were obtained for all study variables. Continuous variables were expressed as medians (25th–75th percentile). Categorical variables were summarized as counts and percentages. Categorical variables were compared using the Fisher exact test or χ^2^-test, as appropriate, and continuous variables were compared using the Mann-Whitney *U*-test. Binomial logistic regression was used to assess the effect of myosteatosis on the likelihood of persistent dyspnea and problems in the EQ-5D dimensions at 6 months after discharge, with age, sex, and BMI as covariates. Multicollinearity was assessed using variance inflation factor (VIF). All VIFs were < 5, ranging between 1.31 and 1.64. All statistical tests were two−tailed, with *p* < 0.05 considered significant. No sample size calculation was performed for the present analysis; the sample size was established by the time window of the study. Missing data were not imputed. Statistical analyses were performed using IBM SPSS Statistics (IBM SPSS Statistics for Windows, Version 22.0. Armonk, NY: IBM Corp.).

## Results

### Patient Characteristics

Between February 25th, 2020 and April 19th, 2020, 652 patients with confirmed SARS-CoV-2 infection were hospitalized at San Raffaele University Hospital, of which 97 underwent a CT scan and had baseline and 6-month clinical and anthropometric data available for analyses (Supplementary Figure 1). Median age was 60.0 years (54.5; 69.5). Most patients were males (79.5%) and of Caucasian ethnicity (88.7%). Overall, nearly one third (32%) of patients had obesity. The most common comorbidity was arterial hypertension (38.5%), followed by DM (10.3%) and coronary heart disease (8.2%). Overall, 17.5% of patients required admission to the ICU during the hospital stay. Median length of stay (LoS) was 15.0 (10.0; 28.0) days.

### Comparison Between Patients With and Without Myosteatosis

**FIGURE 1:**
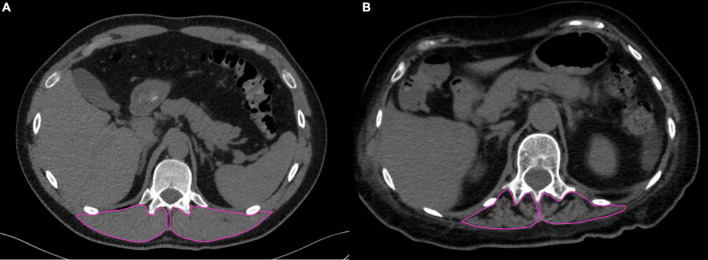
Chest CT images at the T12 level in two patients with COVID-19 pneumonia. In **(A)** (30-year-old man), the skeletal muscle area segmented at the T12 level is characterized by normal mass and high mean SM-RA (56.5 HU); in **(B)** (72-year-old woman), impaired muscle status is evident, with fat infiltration and low mean SM-RA (11.1 HU).

**FIGURE 2:**
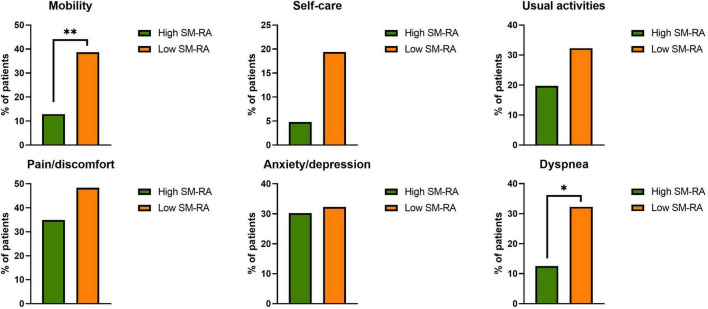
Proportion of patients with any problems in the EQ-5D dimensions (mobility, self-care, usual activities, pain/discomfort, anxiety/depression) and proportion of patients with dyspnea in the group without (high skeletal muscle radiation attenuation, SM-RA) or with (low SM-RA) myosteatosis. *p = 0.021; ^**^p = 0.004.

Median time from admission to CT scan was 3.0 (1.0; 5.5) days. SM-RA cut-offs for defining myosteatosis were 48.90 and 49.98 HU in females and males younger than 60 years, and 18.3 and 36.5 HU in females and males aged 60 years or older, respectively. Figure 1 shows the cross-sectional CT images of a 30-year-old man with high SM-RA and a 72-year-old woman with low SM-RA. A comparison between patients with or without myosteatosis is provided in Table 1. Except for the lymphocyte count, which was significantly lower, and a tendency toward a higher prevalence of obesity in those with myosteatosis, patient characteristics relative to the hospital stay were similar between groups. There was no difference in the proportion of patients admitted to the ICU. By design, SM-RA was significantly lower in patients with myosteatosis. After a median of 184.0 (176.0; 192.0) days since discharge, median BMI and the prevalence of obesity were significantly higher in patients with myosteatosis, as was the proportion of patients with abdominal obesity (Table 2). Although self-rated health (EQ VAS) was similar between groups, in the group with myosteatosis there were more patients complaining of dyspnea and mobility problems, as compared with participants without myosteatosis (Figure 2). The proportion of patients reporting issues in the other EQ5D domains was numerically greater in the group with myosteatosis, the difference in the self-care dimension being borderline significant (19.4 vs. 4.8%, *p* = 0.055).

**TABLE 1:** Comparison between patients with (low SM-RA) or without (high SM-RA) myosteatosis during acute COVID-19.

Variable	High SM-RA (*n* = 66)	Low SM-RA (*n* = 31)	*p*-value
Age, years	60.5 (54.8; 69.3)	60.0 (53.0; 70.0)	0.932
Female sex, *n* (%)	14 (21.2)	6 (19.4)	0.833
Smoke, *n* (%)	25 (39.1)	17 (54.8)	0.147
Ethnicity, *n* (%) Non-Hispanic Hispanic	57 (86.4) 9 (13.6)	29 (93.5) 2 (6.5)	0.498
BMI, kg/m^2^	26.9 (24.8; 30.3)	29.2 (25.2; 33.8)	0.083
Obesity, *n* (%)	17 (25.8)	14 (45.2)	0.056
PaO_2_/FiO_2_	275.2 (167.1; 316.7)	278.6 (232.3; 328.6)	0.333
Length of stay, days	14.0 (8.8; 27.8)	19.0 (11.0; 29.0)	0.161
Temperature,°C	38.0 (37.5; 38.8)	38.3 (36.9; 38.6)	0.820
CRP, mg/Dl	78.4 (31.6; 131.2)	75.7 (31.0; 136.2)	0.772
LDH, U/L	399.5 (308.5; 489.5)	359.0 (280.0; 419.0)	0.202
Plasma glucose, mg/dL	107.0 (99.0; 122.0)	109.5 (99.5; 145.3)	0.566
Hemoglobin, g/dL	14.3 (13.3; 15.3)	14.4 (12.9; 15.2)	0.708
Neutrophil count, ×10^9^/L	4.6 (3.5; 7.5)	5.1 (3.8; 6.9)	0.752
Lymphocyte count, ×10^9^/L	0.8 (0.5; 1.2)	1.0 (0.8; 1.5)	0.046
Creatinine, mg/dL	1.02 (0.83; 1.19)	1.08 (0.86; 1.28)	0.317
eGFR, ml/min/1.73 m^2^	83.6 (69.7; 94.4)	75.5 (62.2; 94.9)	0.387
Platelets, ×10^3^/mm^3^	195.0 (155.0; 235.0)	202.0 (155.0; 232.0)	0.964
Arterial hypertension, *n* (%)	23 (35.4)	14 (45.2)	0.357
Diabetes mellitus, *n* (%)	6 (9.1)	4 (12.9)	0.722
Coronary artery disease, *n* (%)	6 (9.1)	2 (6.5)	1.000
Chronic kidney disease, *n* (%)	2 (3.0)	3 (9.7)	0.167
COPD, *n* (%)	0 (0.0)	2 (6.5)	0.100
Malignancy, *n* (%)	2 (3.0)	0 (0.0)	1.000
Admission to ICU, *n* (%)	10 (15.2)	7 (22.6)	0.463
Time from admission to CT, days	3.0 (1.8; 5.0)	3.0 (1.0; 7.0)	0.322
SM-RA, HU	49.9 (43.2; 53.1)	35.6 (21.4; 44.2)	<0.001
pSMI (cm^2^/m^2^)	8.5 (7.1; 11.4)	9.7 (6.2; 10.8)	0.705

*Continuous variables are expressed as median (25th and 75th percentile). Categorical variables are expressed as absolute values (%). BMI, body mass index; CRP, C-reactive protein; COPD, chronic obstructive pulmonary disease; CT, computed tomography; eGFR, estimated glomerular filtration rate; ICU, intensive care unit; LDH, lactate dehydrogenase; SM-RA, skeletal muscle radiation attenuation; pSMI, paravertebral skeletal muscle index. Percentages are calculated on the actual number of cases.*

**TABLE 2:** Comparison between patients with (low SM-RA) or without (high SM-RA) myosteatosis at 6 months after discharge.

Variable	High SM-RA (*n* = 66)	Low SM-RA (*n* = 31)	*p-*value
Time from discharge, days	186.0 (179.0; 193.0)	183.0 (174.0; 191.0)	0.213
SBP, mmHg	40.0 (130.0; 150.0)	140.0 (130.0; 146.0)	0.641
DBP, mmHg	80.0 (75.0; 86.5)	80.0 (70.0; 86.0)	0.401
Capillary blood glucose, mg/dL	97.0 (90.0; 115.3)	99.0 (92.0; 112.0)	0.477
BMI, kg/m^2^	26.6 (24.6; 30.3)	29.7 (25.8; 35.4)	0.021
Obesity, *n* (%)	18 (27.7)	15 (48.4)	0.046
Waist circumference, cm	101.0 (95.0; 107.0)	109.0 (95.8; 124.3)	0.004
Abdominal obesity, *n* (%)	30 (47.6)	24 (80.0)	0.003
VAS, mm	80.0 (75.0; 90.0)	75.0 (50.0; 90.0)	0.156

*Continuous variables are expressed as median (25th and 75th percentile). Categorical variables are expressed as absolute values (%). BMI, body mass index; SBP, systolic blood pressure; DBP, diastolic blood pressure; VAS, visual analog scale.*

### Effect of Myosteatosis at Hospitalization on Patient-Reported Outcomes at 6 Months After Discharge

To identify predictors of persistent dyspnea at 6 months, we compared anthropometrics and clinical characteristics relative to hospital stay between patients with or without persistent dyspnea. Myosteatosis was significantly more prevalent in those with vs. those without dyspnea (55.6 vs. 27.3%, respectively; *p* = 0.021), as was obesity (61.1 vs. 28.9%, respectively; *p* = 0.01). Consistently, patients with persistent dyspnea had significantly lower SM-RA (40.4 [33.6; 47.3] vs. 47.4 [37.7; 51.6] HU in those with vs. those without dyspnea, respectively; *p* = 0.027), while there was no difference in pSMI (8.9 [7.3; 11.2] vs. 8.9 [7.1; 11.3] in those with vs. those without dyspnea, respectively; *p* = 0.447). There were no between-group differences in age (60.5 [51.0; 68.0] vs. 60.0 [55.0; 70.5] years in those with vs. those without dyspnea, respectively; *p* = 0.456), LoS (15.5 [8.0; 42.8] vs. 15.0 [10.0; 28.0] days, *p* = 0.924) nor in the proportion of males (66.7 vs. 81.8%, *p* = 0.199), history of arterial hypertension (50.0 vs. 36.8%, *p* = 0.304), coronary heart disease (16.7 vs. 6.5%, *p* = 0.172), chronic kidney disease (5.6 vs. 5.2%, *p* = 1.00), DM (11.1 vs. 10.4%, *p* = 1.00), or admission to ICU (22.2 vs. 16.9%, *p* = 0.733). At univariable binomial logistic regression analyses, only myosteatosis was significantly associated with persistent dyspnea at 6 months. Specifically, patients with myosteatosis had threefold the odds to exhibit persistent dyspnea than patients without myosteatosis (OR 3.33 [95% C.I. 1.16; 9.59], *p* = 0.025). This association remained significant after adjustment for sex, age, and BMI (OR 3.19 [95% C.I. 1.04; 9.87], *p* = 0.043).

Similarly, myosteatosis was more prevalent in patients with vs. those with no mobility problems (60.0 vs. 26.0%, *p* = 0.004). Patients with myosteatosis had fourfold the odds to exhibit mobility problems than patients without myosteatosis (OR 4.26 [95% C.I. 1.51; 12.02], *p* = 0.006. This association remained significant after adjustment for sex, age and BMI (OR 3.70 [95% C.I. 1.25; 10.95], *p* = 0.018). There were no other significant between-group differences in anthropometrics and clinical characteristics during acute COVID-19.

There was no significant effect of myosteatosis at hospitalization on other EQ-5D dimensions.

## Discussion

This is the first analysis exploring the association of myosteatosis with patient-reported outcomes in COVID-19 survivors 6 months after discharge. We showed that myosteatosis, as defined by CT-derived SM-RA during acute COVID-19, significantly predicts persistent dyspnea and mobility problems at 6 months. Our results are consistent with those by van Gassel et al., who reported that myosteatosis, rather than muscle mass, was associated with functional impairment at 3 months after discharge in patients who survived ICU admission for COVID-19 (41). These findings, although novel in the context of COVID-19, are not surprising. In fact, fat infiltration may alter muscle architecture and affect muscle function and strength, increasing the risk of mobility problems (12). In addition, ectopic fat deposition in muscle leads to lipotoxicity, which may contribute to the chronic inflammation associated with obesity (42) and to loss of muscle function and mass (43). Sarcopenia, i.e., reduced muscle function and mass, is associated with reduced pulmonary function in community-dwelling adults (44, 45). Low CT-derived trunk SM-RA is associated with worse physical function and dyspnea in patients with non-small cell lung carcinoma (46), with greater severity of thoracic kyphosis (47), which in turn may impair ventilatory mechanics, and with higher postural sway and worse muscle strength and physical function in community-dwelling adults (24, 48). Although SM-RA may differ depending on the muscle considered (49), the finding that myosteatosis in trunk muscles was associated with impaired physical function in previous studies suggests that SM-RA at this site reflects myosteatosis in other muscles, and is consistent with the association between myosteatosis and mobility problems observed in our cohort.

We found that the prevalence of obesity upon admission was numerically greater in patients with vs. those without myosteatosis (45.2 vs. 25.8%, *p* = 0.056), this difference reaching statistical significance at the 6-month follow-up (48.4 vs. 27.7%, *p* = 0.046). Abdominal obesity was also more prevalent among patients with baseline myosteatosis (80.0 vs. 47.6%, *p* = 0.003). Myosteatosis is strongly associated with obesity, and often precedes the development of type 2 diabetes and the metabolic syndrome (50). Persistent inflammation from both obesity and prior SARS-CoV-2 infection may contribute to the long-term sequelae of COVID-19 (51). Of note, however, the association of myosteatosis with dyspnea and mobility problems was independent of BMI, suggesting that fat infiltration of skeletal muscle *per se* contributes to these symptoms. Pre-existing myosteatosis may influence long-term outcomes of COVID-19 survivors causing alterations in muscle architecture and lipotoxicity that may impair muscle function. As in a vicious cycle, pre-existing myosteatosis may lead to further worsening of muscle quality. It has been reported that patients with myosteatosis are at increased risk of muscle loss (52). In the setting of COVID-19 this is highly relevant, as acute sarcopenia, i.e., a decline in muscle mass and muscle function within 28 days of a significant physiological stressor event such as an acute illness (53), is emerging as a clinically relevant consequence of SARS-CoV-2 infection (54–56). This may be particularly true in COVID-19 patients more at risk of myosteatosis, i.e., those with overweight or obesity, who exhibit rapid and wide fluctuations in weight that may worsen body composition (57). Factors contributing to muscle wasting in COVID-19 include direct myotoxicity by SARS-CoV-2, systemic inflammation, bed rest, decreased food intake and myotoxic medications (58). Although we did not assess the effect of COVID-19 on muscle quality, we speculate that patients with prior myosteatosis might be more prone to acute sarcopenia, which in turn might contribute to poorer short-term outcomes and slower functional recovery (41, 59–61).

In contrast with other authors who investigated the relationship between opportunistic chest CT-derived SM-RA and short-term outcomes in COVID-19 (28, 62), we did not find an association between myosteatosis in paraspinal muscles and disease severity as reflected by the need for intensive care, which was similar between patients with or without myosteatosis in our cohort. Giraudo et al. have reported that lower CT-derived SM-RA (< 30 HU) predicts ICU admission in COVID-19 patients (62). However, despite patients with lower SM-RA being significantly older, analyses were not adjusted by age. Age (especially > 60 years) has a dramatic effect on COVID-19 severity (63), as well as on myosteatosis, paraspinal muscles being affected more than other muscles (64). It is difficult to argue that myosteatosis increases the risk of ICU based on the analysis by Giraudo et al., the increase in risk being probably due to age differences. The fat content of skeletal muscle is also affected by sex, with women exhibiting lower SM-RA (greater myosteatosis) than men (49, 64, 65). The use of sex- and age-specific cut-offs for defining myosteatosis is a strength of our analysis. Yi et al. found significant associations between measurements of myosteatosis including SM-RA and transition from mild to severe COVID-19 (28). Differences with our findings may be due to study-specific cut-offs for defining myosteatosis, and different patient characteristics (e.g., older age of our cohort). Other authors investigated the relationship between opportunistic chest CT-derived SM-RA and survival using adjustment for several potential confounders including sex and age, reporting significant associations between thoracic myosteatosis and reduced survival in COVID-19 patients (25, 26).

We also calculated pSMI, which is a biomarker of muscle mass (36, 37). In agreement with previous studies showing that CT-derived muscle density is more strongly associated with muscle function than muscle size (24), even in COVID-19 survivors (41), we found that SM-RA, but not pSMI, was associated with dyspnea and mobility problems at 6 months after discharge. This finding highlights the need of assessing muscle quality, in addition to muscle mass.

We must acknowledge some limitations. The study population consisted of patients who visited our COVID-19 outpatient clinic for the 6-month follow-up, which is prone to selection bias and limited generalizability. Direct CT quantification of muscle fat infiltration, which is an additional marker of muscle quality (66), was not available, and patients did not undergo magnetic resonance imaging (MRI) nor measurement of muscle strength for deeper characterization of muscle quality and function. It should be acknowledged, however, that SM-RA is widely recognized as a biomarker of fat infiltration; it is strongly correlated with muscle fat infiltration measured by MRI-derived proton density fat fraction, and is strongly associated with muscle function (14, 67). The lack of a reference measurement and the relatively small sample size of our cohort prevented us from providing SM-RA cut-offs to univocally diagnose myosteatosis in clinical practice. However, the purpose of our analysis was to describe the relationship between myosteatosis and post-discharge outcomes in COVID-19 survivors. Providing cut-off values for the diagnosis of myosteatosis would require a much larger cohort representative of the general population, and the inclusion of healthy subjects.

## Conclusion

In conclusion, we showed that myosteatosis detected during acute COVID-19 significantly predicts persistent dyspnea and mobility problems at 6 months after hospital discharge. SM-RA can be directly derived from PACS during imaging reporting, and could be easily applied in clinical practice. Given the potential relevance of opportunistic CT-derived SM-RA in detecting patients at risk of persistent functional impairment after COVID-19, larger studies are needed to identify univocal sex- and age- specific cut-offs for diagnosing myosteatosis and promptly implement interventions aimed at improving muscle health and body composition in COVID-19 survivors, to prevent or minimize the long-term sequelae of the disease.

## Data Availability Statement

The raw data supporting the conclusions of this article will be made available by the authors upon reasonable request.

## Ethics Statement

The studies involving human participants were reviewed and approved by the Comitato Etico IRCCS Ospedale San Raffaele. The patients/participants provided their written informed consent to participate in this study.

## Author Contributions

CC, AE, MM, and PR-Q: conception and design of the work. RD, AP, CG, VN, RL, DV, EF, MF, MC, CM, SM, and GV: acquisition of data. CC and RD: analysis of data. CC, AE, MM, PR-Q, AM, RD, and AP: interpretation of data. All authors have approved the submitted version of the manuscript, and have agreed to be personally accountable for the content of the work.

## Conflict of Interest

The authors declare that the research was conducted in the absence of any commercial or financial relationships that could be construed as a potential conflict of interest.

## Publisher’s Note

All claims expressed in this article are solely those of the authors and do not necessarily represent those of their affiliated organizations, or those of the publisher, the editors and the reviewers. Any product that may be evaluated in this article, or claim that may be made by its manufacturer, is not guaranteed or endorsed by the publisher.
