# Supplementary figures and images for: Myosteatosis Significantly Predicts Persistent Dyspnea and Mobility Problems in COVID-19 Survivors
Source: Front Nutr. 2022 Apr 8;9:846901. doi: 10.3389/fnut.2022.846901 (PMC9024358; doi:10.3389/fnut.2022.846901)

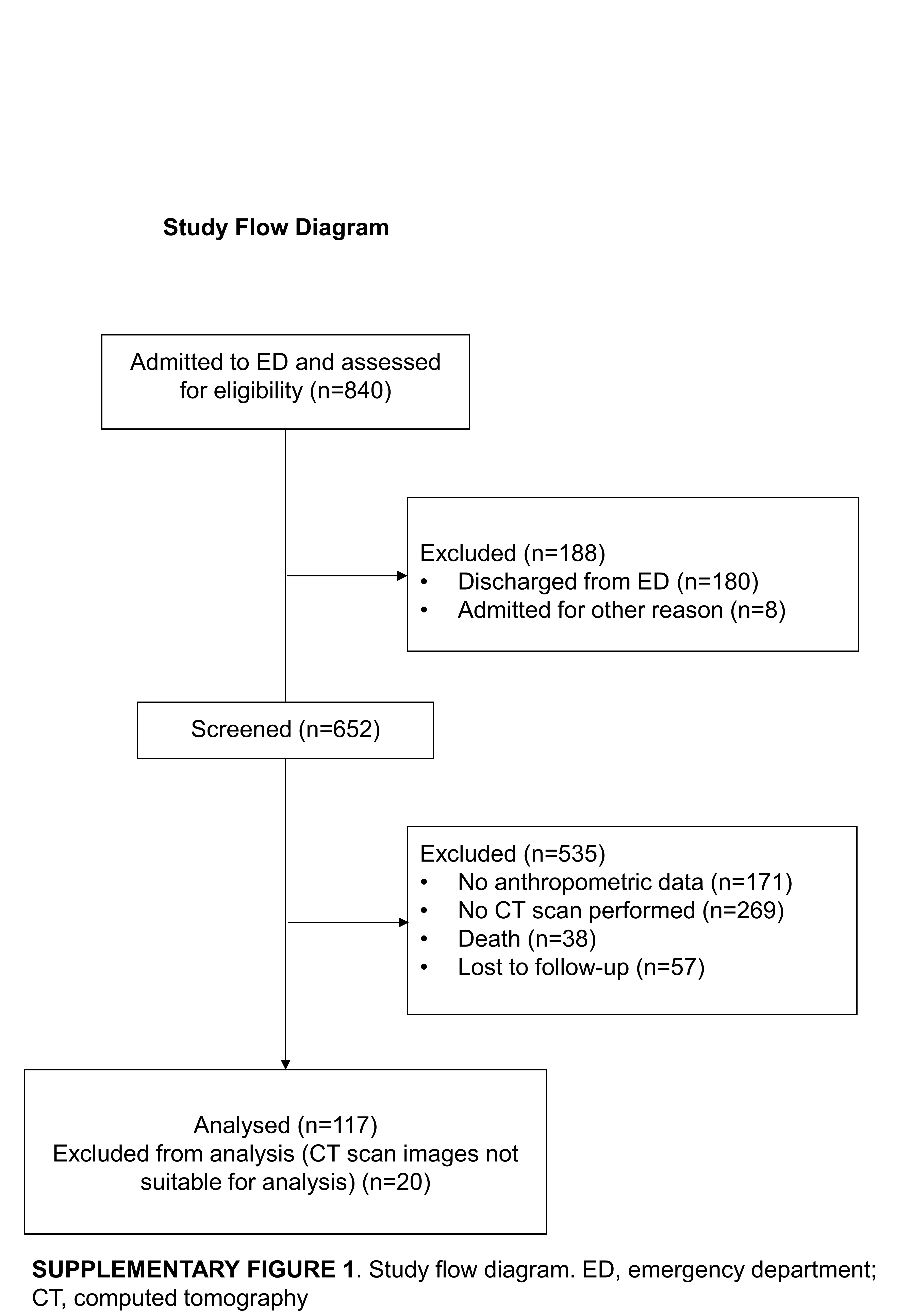

Supplement: Supplementary file 1 [file Image_1.jpg]
